# A case of torsioned giant mucinous cystadenoma mimicking mesenteric cyst

**DOI:** 10.1093/jscr/rjaf180

**Published:** 2025-04-03

**Authors:** Hassen Mohammed Areys, Fitsum Tesfamariam Waldehawariyat, Osman Ali Osman, Abdulhafiz Idris Ibrahim, Isak Omer Ansar

**Affiliations:** Department of Obstetrics and Gynecology, Institute of Health Sciences, Jigjiga University, PO Box 1020, Jigjiga, Ethiopia; Department of Surgery, Institute of Health Sciences, Jigjiga University, PO Box 1020, Jigjiga, Ethiopia; Health System Innovation and Quality Improvement Directorate, Institute of Health Sciences, Jigjiga University, PO Box 1020, Jigjiga, Ethiopia; Department of Radiology, Institute of Health Sciences, Jigjiga University, PO Box 1020, Jigjiga, Ethiopia; Department of Pathology, Institute of Health Sciences, Jigjiga University, PO Box 1020, Jigjiga, Ethiopia

**Keywords:** torsion, giant cyst, ovarian mucinous cystadenoma, mesenteric cyst, case report

## Abstract

Ovarian mucinous cystadenomas are cystic neoplasms lined by mucin-producing epithelial cells. They account for ~15%–20% of ovarian tumors, and in 80% of the cases, they are benign. Intra-abdominal mucinous cystic neoplasms commonly arise from the ovaries but can rarely arise from the mesentery. Here, we report a case of a 49-year-old para II mother who presented with a complaint of progressive abdominal swelling and discomfort of 6 months duration. She had an ill-defined mass around the peri-umbilical and lower abdominal area. Abdominopelvic ultrasound suggested a mesenteric cyst, while computed tomography showed a large, thick-walled cystic lesion of ovarian origin. Determining the tissue of origin of a giant cyst that involves both the mesentery and adnexa is difficult. Torsion of a massive ovarian cyst is a rare gynecologic emergency that involves both diagnostic and management challenges. No matter the diagnostic dilemmas, surgery is the mainstay of treatment.

## Introduction

Ovarian mucinous cystadenomas (MCA) are cystic neoplasms lined by mucin-producing epithelial cells. It’s a multilocular cyst with smooth exterior and inner surfaces. It accounts for ~15%–20% of ovarian tumors. In 80% of the cases, it is benign [1–3]. The tumor is usually unilateral but becomes bilateral in 5%–10% of cases. Ovarian cysts rarely grow immensely; however, cysts >10 cm in diameter are often called “giant” ovarian cysts [4–6]. They usually occur between the fourth and fifth decades of life, even though they are sporadic at extreme ages [7, 8]. Intra-abdominal mucinous cystic neoplasms commonly arise from the ovaries, but they can rarely arise from the pancreas, liver, spleen, or mesentery. Determining the tissue of origin of giant cysts that involve both the mesentery and adnexa is difficult [9]. Ovarian MCAs are usually asymptomatic at early stages; however, giant cysts present with mass-effect symptoms [3, 10].

The most frequent complications of a benign ovarian cyst are torsion, hemorrhage, and rupture [3, 5]. Torsion of an ovarian cyst is a gynecologic emergency that usually presents as an acute abdominal condition, with the typical presentation being sudden severe ipsilateral lower abdominal pain that worsens with a change in position and tender mass upon physical examination. It occurs when the tumor pedicle is long, the tumor has good mobility, and the center of gravity is shifted to one side, with an incidence rate of 10% [11, 12].

Torsion of a giant benign ovarian cyst is rare and often involves diagnostic and management challenges. Accurate and timely diagnosis and surgical intervention can prevent further irreversible damage to the ovary and preserve future fertility. Abdominopelvic ultrasound scanning can be used as an early diagnostic modality. A computed tomography (CT) scan is also used to show the consistency and the level of tumor extension [4, 10, 13]. Management of an ovarian cyst depends on the patient’s age, the size of the cyst, its histopathological nature, and the presence of complications. Conservative surgical intervention such as ovarian cystectomy and salpingo-oophorectomy is adequate for benign ovarian lesions [6, 10].

Here, we report a case of torsioned giant ovarian mucinous cystadenoma that mimicked a mesenteric cyst. The cyst measures 19.6 × 17.6 × 13 cm, making it the largest ovarian cyst in Jigjiga City, Somali Region, Ethiopia.

## Case presentations

A 49-year-old para II mother presented at the surgical outpatient clinic with a complaint of progressive abdominal swelling and discomfort of 6 months duration. Currently, she has presented with diffuse abdominal pain for the last 24 hours. Her menarche commenced at the age of 14 years. The patient had no previous medical diseases, medication usage, or surgical operations. On physical examination, she was in pain, her blood pressure was 100/60 mmHg with a pulse rate of 116 beats/minute, her respiratory rate was 24 cycles/minute, and her axillary temperature was 36.5°C. On abdominal examination, there was an ill-defined mass that moved side to side, not fixed to the underlying tissue, with mild tenderness in the peri-umbilical area and the lower abdomen. There was no shifting dullness.

**Figure 1:**
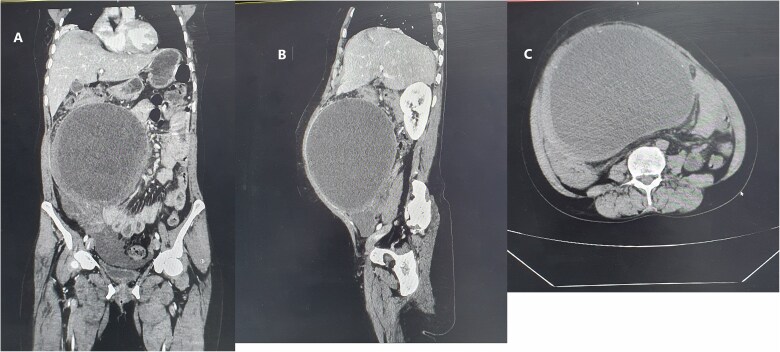
Homogenous hypoattenuating large unilocular cystic mass occupying abdominopelvic cavity with some areas of high attenuating of different viscosity and ovarian vascular pedicle sign as an organ of origin measuring 19.6 × 17.6 × 13 cm (green arrow) (A) coronal enhanced abdominal CT scan (B) enhanced sagittal abdominal CT scan (C) non enhanced upper abdomen axial CT scan.

Laboratory parameters were in the normal range. Abdominopelvic ultrasound suggested an intraabdominal cyst that extends to the pelvis with small free fluid collection adjacent to the cyst. Abdominopelvic computed tomography with contrast showed a large, thick-walled mesenteric cystic lesion measuring 19.6 × 17.6 × 13 cm, likely of ovarian origin. The cyst occupied the right upper and lower abdominal quadrants, crossing the midline. It extends from the subhepatic region at the T12 to the S1 level of the vertebrae (Fig. 1). Since the organ of origin was difficult to settle, the gynecology department was consulted to overview the case and jointly manage the patient.

**Figure 2:**
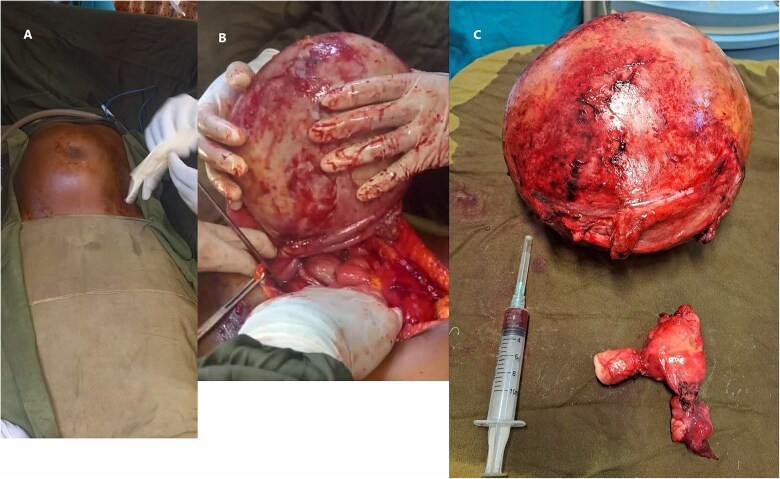
Shows torsion of the right ovarian cystic mass (A) shows the abdominal mass before the surgical excision (B) shows the 360° counterclockwise twist of the right adnexal cyst (green arrow) (C) the resected right adnexal mass along with the uterus.

**Figure 3:**
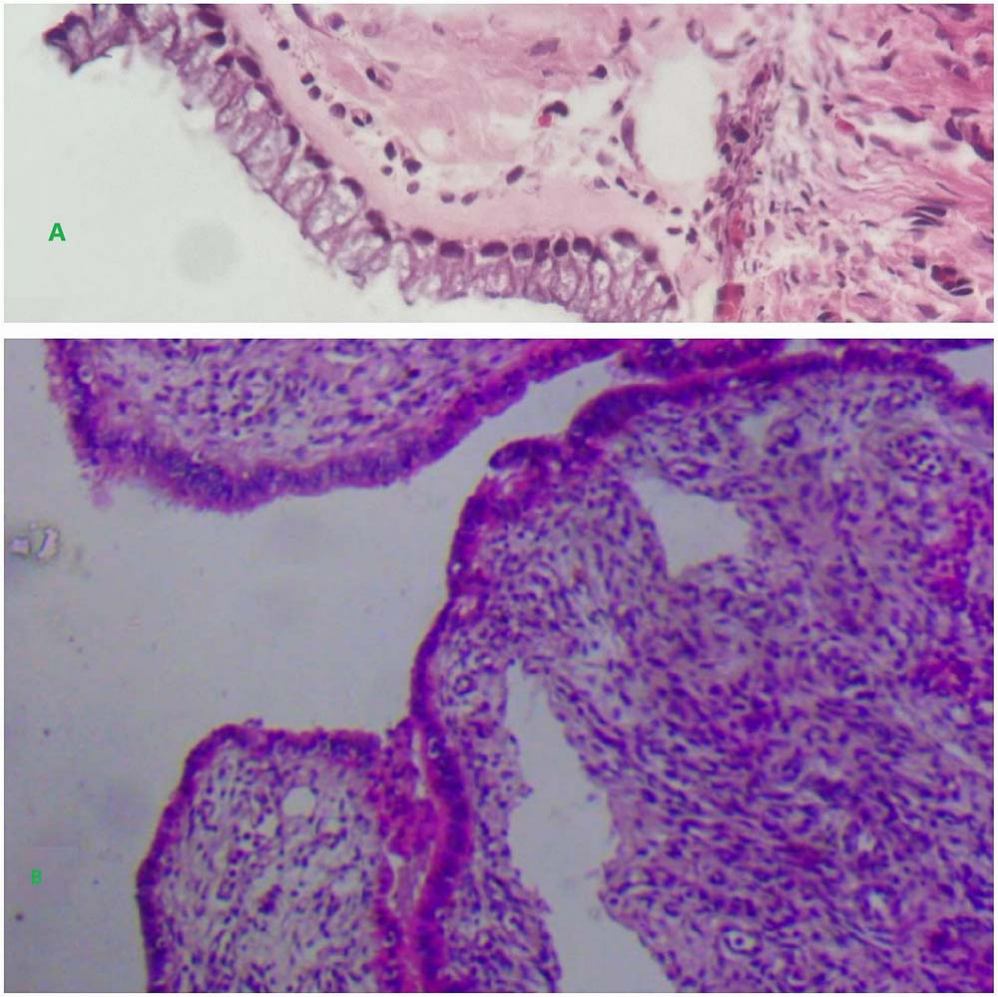
Shows a unilocular cyst with glands lined by a single bland appearing columnar to cuboidal mucinous epithelium.

The patient was then counseled about the procedure and signed informed consent for surgical exploration. Under general anesthesia, an exploratory laparotomy was done with intraoperative findings of a huge central abdominal cystic mass arising from the right adnexa with a moment of torsion 360° counterclockwise at the tube. The cystic mass was resected together with the right adnexa, and then a total abdominal hysterectomy (TAH) plus bilateral salpingo-oophorectomy (BSO) was done (Fig. 2). The resected sample was then sent for pathology analysis. The pathology report described a fibrous cystic wall, lined with columnar cells containing intracellular mucin, with no feature of malignancy, compatible with benign mucinous cystadenoma (Fig. 3). The patient had a smooth recovery and was discharged on her 5^th^ postoperative day.

## Discussion

Ovarian epithelial tumors are primarily cystic, with papillary projections as a distinctive feature. They are classified as serous and mucinous tumors, endometrioid and clear cell carcinomas, and Brenner tumors [1–3]. In this case, the patient was diagnosed with ovarian MCA. The peak incidence of ovarian mucinous cystadenomas usually occurs between the fourth and fifth decades of life [7, 8]. In this case, the patient was in her 5^th^ decade of life. In our case, the ovarian MCA was unilateral, consistent with most presentations.

Intraabdominal ovarian MCAs are usually asymptomatic and may be detected incidentally. Giant abdominal masses can compress local organs and lead to obstruction and sometimes potential necrosis of these organs [6]. In this case, the patient presented with progressively increasing abdominal swelling, diffuse abdominal pain and tenderness, tachycardia, and a giant mass. Benign ovarian cysts usually have either torsion, hemorrhage, or rupture as a major complication. Torsion of a giant benign ovarian tumor is a rare complication that occurs when the tumor pedicle is long and its center of gravity is shifted to one side [6, 11, 12]. In this case, the patient presented with an ovarian torsion. MCAs of the ovary are known for their potential to grow massively; in this case, the ovarian MCA measured 19.6 × 17.6 × 13 cm.

Large intra-abdominal cystic lesions can present with certain diagnostic challenges and difficulties due to image overlapping of different abdominal entities. Cysts from the mesentery and omentum may mimic those of adnexal masses. Abdominopelvic ultrasound scanning as an early diagnostic modality and CT scan to show the consistency and level of extension of the tumor is used. Further surgical exploration might be required to confirm the origin and pathology of the cyst [6, 13, 14]. In our case, since the mass was giant, the abdominopelvic ultrasound suggested a mesenteric cyst; while the computed tomographic scan suggested an intraabdominal cystic mass likely of ovarian origin. The pathology report then showed benign ovarian mucinous cystadenoma. The laparotomy findings and the results of the histopathological examination confirmed the origin and diagnosis of the tumor.

Management of ovarian cysts depends on the patient’s age, menopausal status, the size and structure of the cyst, and the presence of complications. Complete surgical enucleation of the cyst is the gold standard approach for the management of a giant chylous cyst. Conservative surgical intervention, cystectomy with salpingo-oophorectomy, is adequate for young patients with benign ovarian lesions. For a woman in her perimenopausal and postmenopausal age, TAH with BSO and tumorectomy are recommended for the risk of malignant transformation. If the patient presents with an acute abdomen, the need for urgent surgery is necessary to avoid irreparable damage [6, 10, 15]. In our case, since the patient was in her fifth decade of life and presented with mega ovarian torsion, the cystic mass was resected together with the right adnexa, and then a TAH plus BSO was done.

## Conclusion

A giant ovarian cyst is a rare gynecologic condition, and management is challenging, mainly because it is difficult to establish the correct diagnosis up front. Regardless of the diagnostic challenges and the origin of the cystic lesion, surgery is the mainstay of treatment to avoid further worsening of the patient’s condition and complications.

## References

[1] Alobaid A, Elamir H, Abuzaid M, et al. An extremely giant ovarian mucinous cystadenoma. Gulf J Oncolog 2019;1:83–6. 30956200

[2] Gorgone S, Minniti C, Ilacqua A, et al. Giant mucinous cystadenoma in a young patient. A case report. Il Giornale di Chirurgia-J Italian Surg Assoc 2008;29:42–4. 18252148

[3] Kamel RM . A massive ovarian mucinous cystadenoma: a case report. Reprod Biol Endocrinol 2010;8:1–3. 10.1186/1477-7827-8-24. 20222970 PMC2848663

[4] de Lima SHM, Dos Santos VM, Darós AC, et al. A 57-year-old Brazilian woman with a giant mucinous cystadenocarcinoma of the ovary: a case report. J Med Case Reports 2014;8:1–5. 10.1186/1752-1947-8-82. PMC3946053 24594205

[5] Posabella A, Galetti K, Engelberger S, et al. A huge mucinous cystadenoma of ovarian: a rare case report and review of the literature. Rare Tumors 2014;6:42–3. 10.4081/rt.2014.5225. PMC4083665 25002945

[6] Pucakoski S, Spiroska N, Nikolovski A. Torsion of a large ovarian cyst presented as an acute abdomen: case report. Archiv Public Health 2022;14:107–12. 10.3889/aph.2022.6062.

[7] Sri Paran T, Mortell A, Devaney D, et al. Mucinous cystadenoma of the ovary in perimenarchal girls. Pediatr Surg Int 2006;22:224–7. 10.1007/s00383-005-1624-1. 16416281

[8] Vizza E, Galati GM, Corrado G, et al. Voluminous mucinous cystadenoma of the ovary in a 13-year-old girl. J Pediatr Adolesc Gynecol 2005;18:419–22. 10.1016/j.jpag.2005.09.009. 16338609

[9] Lambert RG, Cervantes BYH, Gonzalez MR, et al. Large intra-abdominal mucinous cystic adenoma: is it of ovarian or mesenteric origin. Pan African Med J 2020;36. 10.11604/pamj.2020.36.122.21642. PMC7422744 32849977

[10] Katke RD . Giant mucinous cystadenocarcinoma of ovary: a case report and review of literature. J Mid-life Health 2016;7:41–4. 10.4103/0976-7800.179167. PMC4832896 27134482

[11] Hongju H . A giant ovarian cyst torsion: case report. Medicine 2024;103:e33283. 10.1097/MD.0000000000033283. 38608053 PMC11018219

[12] Spinelli C, Piscioneri J, Strambi S. Adnexal torsion in adolescents: update and review of the literature. Curr Opin Obstetr Gynecol 2015;27:320–5. 10.1097/GCO.0000000000000197. 26204167

[13] Jung SE, Lee JM, Rha SE, et al. CT and MR imaging of ovarian tumors with emphasis on differential diagnosis. Radiographics 2002;22:1305–25. 10.1148/rg.226025033. 12432104

[14] Boob MM, Chavan NN, Kapote D, et al. An interesting case of an adnexal mass mimicking a mesenteric cyst in pregnancy. J South Asian Federation Obstetr Gynaecol 2023;15:248–50. 10.5005/jp-journals-10006-2209.

[15] Isaia M, Erodotou M, Nakos G, et al. Complete surgical enucleation of a giant chylous mesenteric cyst. Case Rep Surg 2020;2020:4279345. 10.1155/2020/4279345. 32257496 PMC7104311

